# Age and sex differences in cause-specific excess mortality and years of life lost associated with COVID-19 infection in the Swedish population

**DOI:** 10.1093/eurpub/ckad086

**Published:** 2023-06-01

**Authors:** Christina E Lundberg, Ailiana Santosa, Jonas Björk, Maria Brandén, Ottmar Cronie, Martin Lindgren, Jon Edqvist, Maria Åberg, Martin Adiels, Annika Rosengren

**Affiliations:** Department of Molecular and Clinical Medicine, Institute of Medicine, Sahlgrenska Academy, University of Gothenburg, Gothenburg, Sweden; Department of Food and Nutrition, and Sport Science, University of Gothenburg, Gothenburg, Sweden; Department of Molecular and Clinical Medicine, Institute of Medicine, Sahlgrenska Academy, University of Gothenburg, Gothenburg, Sweden; School of Public Health and Community Medicine, Institute of Medicine, Sahlgrenska Academy, University of Gothenburg, Gothenburg, Sweden; Division of Occupational and Environmental Medicine, Department of Laboratory Medicine, Faculty of Medicine, Lund University, Lund, Sweden; Clinical Studies Sweden, Forum South, Skåne University Hospital, Lund, Sweden; Stockholm University Demography Unit (SUDA), Department of Sociology, Stockholm University Demography Unit, Stockholm University, Stockholm, Sweden; Institute for Analytical Sociology, Linköping University, Norrköping, Sweden; Department of Mathematical Sciences, Chalmers University of Technology and University of Gothenburg, Gothenburg, Sweden; Department of Molecular and Clinical Medicine, Institute of Medicine, Sahlgrenska Academy, University of Gothenburg, Gothenburg, Sweden; Department of Medicine Geriatrics and Emergency Medicine, Sahlgrenska University Hospital Östra Hospital, Region Västra Götaland, Gothenburg, Sweden; Department of Molecular and Clinical Medicine, Institute of Medicine, Sahlgrenska Academy, University of Gothenburg, Gothenburg, Sweden; School of Public Health and Community Medicine, Institute of Medicine, Sahlgrenska Academy, University of Gothenburg, Gothenburg, Sweden; Regionhälsan, Region Västra Götaland, Gothenburg, Sweden; School of Public Health and Community Medicine, Institute of Medicine, Sahlgrenska Academy, University of Gothenburg, Gothenburg, Sweden; Department of Molecular and Clinical Medicine, Institute of Medicine, Sahlgrenska Academy, University of Gothenburg, Gothenburg, Sweden; Department of Medicine Geriatrics and Emergency Medicine, Sahlgrenska University Hospital Östra Hospital, Region Västra Götaland, Gothenburg, Sweden

## Abstract

**Background:**

Estimating excess mortality and years of life lost (YLL) attributed to coronavirus disease 19 (COVID-19) infection provides a comprehensive picture of the mortality burden on society. We aimed to estimate the impact of the COVID-19 pandemic on age- and sex-specific excess mortality and YLL in Sweden during the first 17 months of the pandemic.

**Methods:**

In this population-based observational study, we calculated age- and sex-specific excess all-cause mortality and excess YLL during 2020 and the first 5 months of 2021 and cause-specific death [deaths from cardiovascular disease (CVD), cancer, other causes and deaths excluding COVID-19] in 2020 compared with an average baseline for 2017–19 in the whole Swedish population.

**Results:**

COVID-19 deaths contributed 9.9% of total deaths (98 441 deaths, 960 305 YLL) in 2020, accounting for 75 151 YLL (7.7 YLL/death). There were 2672 (5.7%) and 1408 (3.0%) excess deaths, and 19 141 (3.8%) and 3596 (0.8%) excess YLL in men and women, respectively. Men aged 65–110 years and women aged 75–110 years were the greatest contributors. Fewer deaths and YLL from CVD, cancer and other causes were observed in 2020 compared with the baseline adjusted to the population size in 2020.

**Conclusions:**

Compared with the baseline, excess mortality and YLL from all causes were experienced in Sweden during 2020, with a higher excess observed in men than in women, indicating that more men died at a younger age while more women died at older ages than expected. A notable reduction in deaths and YLL due to CVD suggests a displacement effect from CVD to COVID-19.

## Introduction

Like many other countries during the coronavirus disease 19 (COVID-19) pandemic in 2020, Sweden experienced a surge in overall deaths, many attributable to infection by severe acute respiratory syndrome coronavirus 2.^1^^,^^2^ Using excess mortality and years of life lost (YLL) to measure the impact of COVID-19 infection may provide a less biased estimate of the mortality burden during the pandemic and a better understanding of the substantial variety of these two metrics by age and sex over time.^3^ In prior studies, evident overall excess mortality during the COVID-19 pandemic has been reported in the USA,^4–7^ Europe,^3^^,^^8–16^ Japan,^17^^,^^18^ Sweden^19^^,^^20^ and several other countries in Europe^8^ experienced excess all-cause mortality during the first months of the pandemic while other countries including Australia, Denmark, Norway and Georgia experienced lower mortality rates.^14^ No sex difference in excess mortality was identified in Brazil, France, Italy, Spain, Sweden, England, Wales, Northern Ireland, Scotland or the USA. In Italy, however, excess deaths were driven by men, whereas in Ireland, only female deaths exceeded expected levels.^14^ As of 2020, life expectancy at birth has declined in most countries, notably among men^7^^,^^21^^,^^22^ and people over 60 years of age.^23^ An analysis of 81 countries indicated that the average number of YLL per death was 16, with three-quarters of YLL attributed to deaths among people below 75 years of age, and with men losing 45% more years of life than women.^24^ As shown in another study, during the first half of 2020, Belgium had the highest number of YLL attributable to COVID-19 infection, followed by the UK, Italy, Sweden and France.^25^

Although the impact of COVID-19 on all-cause mortality has been widely studied, separating the contributions to excess mortality and YLL of various causes of death is extremely important. How mortality from causes other than COVID-19 has been affected by the pandemic and the extent to which mortality has varied by sex and age remains unclear. The results of a recent study conducted on the older Swedish population showed YLL-related COVID-19 in 2020, after adjustment for shorter life expectancy due to the need for care in older more frail individuals, was comparable to the YLL from ischaemic heart disease in 2019 and 2020.^26^ As of yet, no studies have quantified the impact of the COVID-19 pandemic on excess mortality and YLL for all-cause death and specific causes of death for the entire Swedish population based on a longer baseline period. Using timely and reliable data from national Swedish register data, this study aims to estimate the impact of the COVID-19 pandemic on age- and sex-specific excess mortality and YLL in Sweden during 2020 and over the first 5 months of 2021 by comparing mortality and YLL in Sweden during this period to that during 2017–19.

## Methods

### Study population and data sources

A population-based observational study of annual data was conducted by linking Swedish administrative and healthcare registries using the unique personal identification number provided to all Swedish citizens. We included all individuals alive on January 1 during 2017, 2018, 2019 and 2020 and registered in the Swedish Registry of the Total Population. At the time of the analysis, data on all-cause-specific deaths provided by the Cause of Death Register were only available until May 2021. Comorbidities and age- and sex-specific annual life expectancy tables for 2017–19 were collected from the National Patient Register and Statistics Sweden, respectively.^27^

The main study outcome was (i) the number of excess deaths during 2020–May 2021 and (ii) YLL during 2020. We estimated the number of excess deaths by comparing the total number of deaths to the average number of deaths reported for the corresponding period over the previous 3-year baseline (2017–19), expressing change in absolute terms and as the percentage of additional deaths in a given period compared with deaths during a baseline period not affected by the pandemic. We extracted 1-year life expectancy tables with remaining life expectancy by age, year (2017–19), and sex from the life tables in Statistics Sweden. The mean life expectancy during the 2017–19 period was used to calculate YLL for 2020. YLL was calculated by summing the number of deaths at each age of death between 0 and 110 years and multiplying this by the number of remaining age-, sex- and year-specific life expectancy. We could not calculate YLL for 2021 because of the lack of available life expectancy information from Statistics Sweden. We calculated YLL using the following formula:


$$\;\mathrm{YLL}\;=\;\sum_{i}M_{i}\;*\;\left({\mathrm{LE}}_{i}-{\mathrm{IRP}}_{i}\right)$$

where *i* denotes the 1-year age group, $M_{i}$ denotes the number of deaths registered in age group *i,* LE denotes remaining life expectancy in age group *i* and IRP denotes the intermediate-range point of the age group *i*.

Excess mortality and YLL were calculated separately for men and women and age groups. On the basis of estimates of total and monthly excess mortality, we calculated the percentage difference between deaths observed in 2020 and average deaths in 2017–19. For YLL, we calculated the annual percentage difference from the average baseline. When calculating the monthly excess mortality, all deaths in 2017–19 were calculated and presented as crude average numbers, without any adjustments. When calculating total excess mortality and YLL, all deaths in 2017–19 were adjusted to the population size in 2020.

To understand the full impact of deaths attributable to COVID-19, we grouped deaths into direct COVID-19 and indirect/non-COVID-19 deaths, i.e. the total number of deaths minus the number of COVID-19 deaths. Deaths due to COVID-19 were defined by the *International Classification of Disease* (ICD) version 10 U07.1 or U07.2 as the underlying cause of death. In addition, we computed the cause-specific number of excess deaths and YLL based on the most common causes of death in Sweden: cardiovascular disease (CVD), cancer (C00–C97) (Supplementary table S1), and other causes (excluding CVD, cancer and COVID-19). Age groups were 0–44, 45–64, 65–74, 75–84 and 85–110 years. Data management and analyses were performed using SAS version 9.4 and R version 4.0.

## Results

Overall and sex-specific-adjusted deaths and YLL for cause-specific deaths are presented in table 1. A detailed comparison of the adjusted number of deaths and YLL between each baseline year (2017–19, the period used to establish life tables for estimating YLL) and 2020 is shown in Supplementary tables S2 and S3. During 2020 and the first 5 months of 2021, 98 441 (table 1) and 40 390 (Supplementary table S4) crude-registered deaths, respectively, were observed in Sweden. Deaths during 2020 accounted for 960 305 YLL (9.8 YLL/death); 50.4% (49 597) of deaths and 54.0% (518 703) of YLL occurred among men. A total of 9745 (9.9%) confirmed deaths were attributed to COVID-19 in 2020, with the majority among men (5274 deaths, 54.1%). COVID-19 deaths accounted for a total of 75 151 YLL (7.7 YLL/death), of which 43 384 YLL (8.4 YLL/death) were in men and 31 768.2 (7.1 YLL/death) were in women (table 1). Those who died from COVID-19 were more likely to be men (54%), and on average 3 years older than those who died from other causes than COVID-19. The mean age of those who died from COVID-19 was higher for women than for men (86.1 ± 9.6 vs. 82.0 ± 10.6 years). Among those who died from COVID-19, 45.9% (48.9% of men and 42.3% of women) had CVD or cancer as contributory causes of death, which was considerably higher than among those who died from causes other than COVID-19 (Supplementary table S5).

**Table 1:** All and cause-specific adjusted^a^ excess deaths, years of life lost and years of life lost per death compared with average number of deaths and years of life lost during 2017–2019, for total and by men and women

	Average baseline 2017–2019			2020			Difference compared with average baseline	
	Deaths (column %)	YLL (column %)	YLL/death	Deaths (column %)	YLL (column %)	YLL/death	Deaths, *n* (%)	YLL, *n* (%)
All								
All deaths	94 361 (100)	937 568 (100)	10.3	98 441 (100)	960 305 (100)	9.8	4080 (8.4)	22 736 (2.4)
CVD	25 091 (26.6)	187 811 (20.0)	7.8	22 366 (22.7)	168 000 (17.5)	7.5	−2725 (−10.9)	−19 811 (−10.5)
Cancer	23 529 (24.9)	292 756 (31.2)	12.9	22 523 (22.9)	278 224 (29.0)	12.4	−1006 (−4.3)	−14 532 (−5.0)
Other	45 740 (48.5)	457 001 (48.7)	10.4	43 807 (44.5)	438 930 (45.7)	10.0	−1933 (−4.2)	−18 071 (−4.0)
COVID-19	NA	NA	NA	9745 (9.9)	75 151 (7.8)	7.7	9745 (NA)	75 151 (NA)
Deaths excluding COVID-19	90 793 (100)	937 568 (100)	10.3	88 696 (90.1)	885 153 (92.2)	10.0	−5665 (−6.0)	−52 415 (−5.6)
Men								
All deaths	46 925 (100)	499 562 (100)	11.2	49 597 (100)	518 703 (100)	10.5	2672 (5.7)	19 141 (3.8)
CVD	12 681 (27.0)	108 211 (21.7)	9.0	11 452 (23.1)	98 280 (18.9)	8.6	−1229 (−9.7)	−9931 (−9.2)
Cancer	12 393 (26.4)	141 529 (28.3)	12.0	11 872 (23.9)	135 557 (26.1)	11.4	−521 (−4.2)	−5972 (−4.2)
Other	21 851 (46.6)	249 822 (50.0)	12.0	20 999 (42.3)	241 483 (46.6)	11.5	−852 (−3.9)	−8339 (−3.3)
COVID-19	NA	NA	NA	5274 (10.6)	43 384 (8.4)	8.2	5274 (NA)	43 383 (NA)
Deaths excluding COVID-19	46 925 (100)	499 562 (100)	11.2	44 323 (89.4)	475 319 (91.6)	10.7	−2602 (−5.5)	−24 242 (−4.9)
Women								
All deaths	47 436 (100)	438 006 (100)	9.5	48 844 (100)	441 602 (100)	9.0	1408 (3.0)	3596 (0.8)
CVD	12 410 (26.2)	79 600 (18.2)	6.6	10 914 (22.3)	69 720 (15.8)	6.4	−1496 (−12.1)	−9880 (−12.4)
Cancer	11 136 (23.4)	151 227 (34.5)	14.0	10 651 (21.8)	142 667 (32.3)	13.4	−485 (−4.4)	−8560 (−5.7)
Other	23 889 (50.4)	207 179 (47.3)	8.9	22 808 (46.7)	197 447 (44.7)	8.7	−1081 (−4.5)	−9732 (−4.7)
COVID-19	NA	NA	NA	4471 (9.2)	31 768 (7.2)	7.1	4471 (NA)	31 768 (NA)
Deaths excluding COVID-19	47 436 (100)	438 006 (100)	9.5	44 373 (90.8)	409 834 (92.8)	9.2	−3063 (−6.5)	−28 172 (−6.4)

a Adjusted to the population size of 2020.

**Figure 1:**
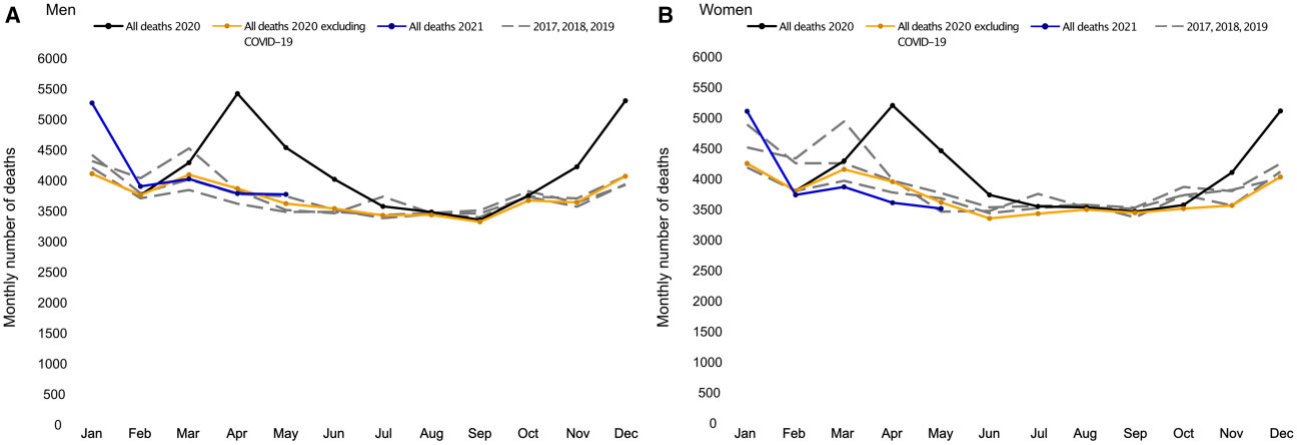
Crude monthly deaths from all**-**causes, deaths excluding COVID-19 during 2020, and all-cause deaths during the first 6 months of 2021, compared with average monthly deaths from all causes during 2017–2019, in A) men and B) women

The crude relative change in all-cause mortality during 2020 compared with that during 2017–19 showed two pronounced peaks; the first appeared in April–June 2020 and the second in November 2020–January 2021 in both men and women. Except for these peaks, the monthly mortality trend in 2020 was similar to that in 2017–19 among both men and women; however, monthly mortality was considerably lower from February to May 2021 among women but not among men (figure 1, Supplementary figure S1 and tables S4 and S6).

**Figure 2:**
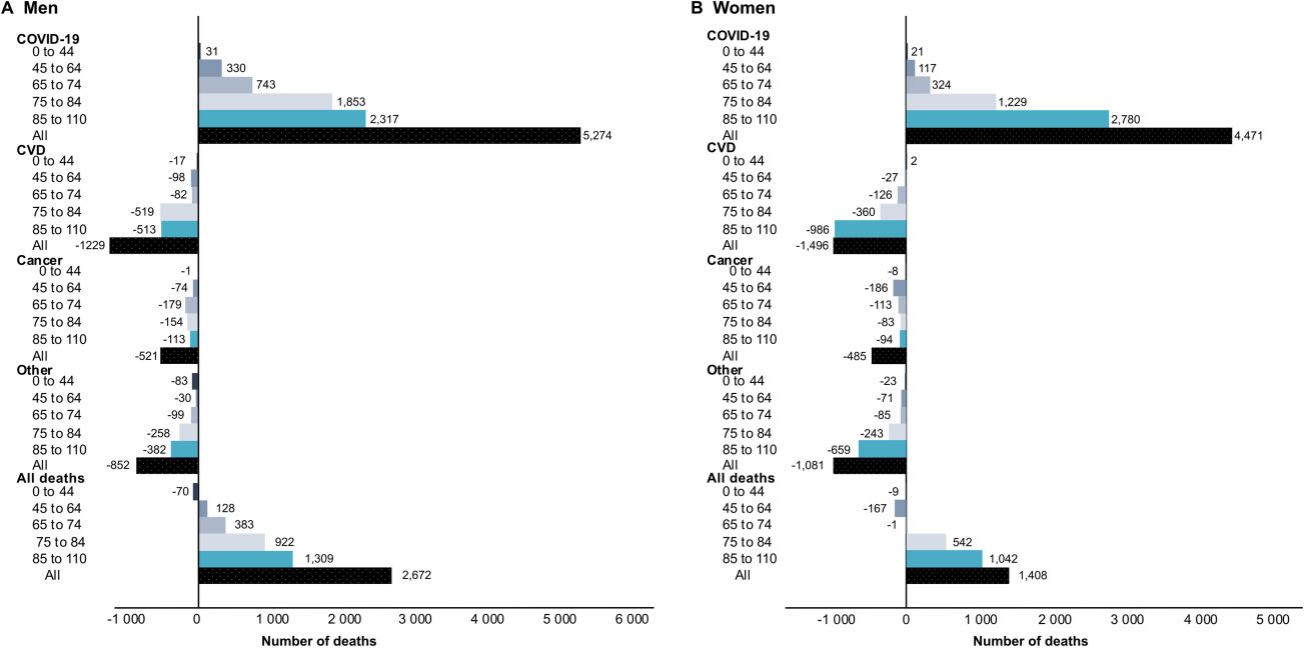
Adjusted excess number of deaths from all causes of deaths and for selected causes of deaths during 2020 compared with average number of death and years of life lost during 2017–2019, by age groups and men (A) and women (B). Adjusted to the population size of 2020

**Figure 3:**
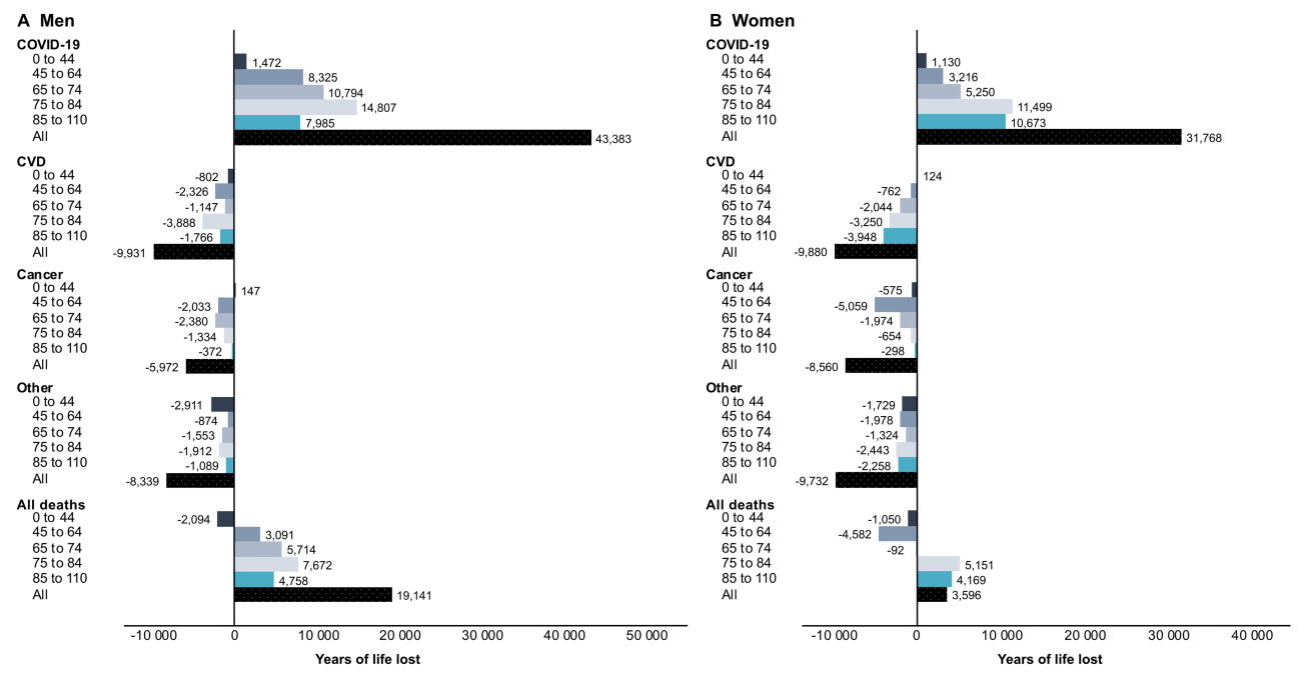
Adjusted excess years of life lost from all causes of deaths and for selected causes of deaths during 2020 compared with average number of death and years of life lost during 2017–2019, by age groups and men (A) and women (B). Adjusted to the population size of 2020

In 2020, 4080 (8.4%) adjusted excess deaths and 22 736 (2.4%) adjusted excess YLL were observed in Sweden compared with the average baseline, 2017–19 (table 1). A majority of the excess deaths occurred among men [2672 (65%) deaths, a relative increase of 5.7%], while 1408 [(35%), a relative increase of 3.0%] occurred among women, resulting in an excess of 19 141 (a relative increase of 3.8%) YLL among men and an excess of 3596 (relative increase of 0.8%) YLL among women (table 1). Excess deaths and YLL were observed in all age groups except the 0–44-year group among men, while excess deaths and YLL in women were only seen in the two oldest age groups (75+ years). Men and women aged 75–84 had the highest number of excess YLL from all-cause deaths (figures 2 and 3, Supplementary figures S2 and S3). A similar trend was observed for COVID-19 deaths in those aged 75 years and older, with 4170 (79.1%) and 4009 (89.7%) deaths among men and women, respectively (figure 2). Furthermore, the highest contribution of excess YLL among men was from COVID-19 deaths in the 65–74- and 75–84-year age groups (10 794 and 14 807 YLL, respectively) accounting for 59% of the excess YLL. Among women, COVID-19 deaths also contributed most to excess YLL, although only among those aged 75 years and older (22 172, 69.8% of total excess YLL; figure 3).

### Cause-specific death

During 2020, a relative decrease in the adjusted number of deaths and YLL from all specific causes of death was observed compared with the average baseline. The magnitude of the decrease varied across causes of death, age and sex. The largest decrease in specific causes was observed in the number of deaths due to CVD among both women (−12.1% deaths and −12.4% YLL) and men (−9.7% deaths and −9.2% YLL; table 1, figures 2 and 3, Supplementary figures S2 and S3). Adjusted deaths and YLL from CVD during 2020 were relatively lower than the average baseline across all age groups in both men and women, with fewer deaths among men and women aged 85+, while the highest reduction in the number of YLL was among men aged 75–84 years and women aged 85+ years. The adjusted number of deaths and YLL due to cancer and other causes during 2020 were also lower compared with the average baseline across all age groups, with fewer deaths and YLL, especially in younger ages for cancer and among those aged 75+ for other causes, in both men and women (figure 2, Supplementary figures S2 and S3). The YLL from cancer and other causes decreased in 2020 compared with the average baseline across all age groups in men and women (figure 3, Supplementary figures S2 and S3). When including all COVID-19 cases with CVD or cancer as a contributing cause of death among CVD and cancer cases, the number of deaths and YLL were similar to the average baseline (Supplementary table S7).

## Discussion

The current study attempts to quantify age- and sex-specific excess mortality and YLL associated with total and specific causes of death in the entire Swedish population during 2020 and the first 5 months of 2021, compared with the period 2017–19. We observed that both men and women experienced excess deaths and YLL from death due to all causes during 2020, but only in the 45+ age group in men and the 75+ age group in women. Excess deaths were predominantly due to COVID-19, with a greater impact on men than on women, especially among those aged 75 years and older. Simultaneously, we observed a considerable decrease in the number of deaths and YLL from CVD for both sexes, predominantly in ages where deaths from COVID-19 were most common. This could potentially indicate a mortality displacement during 2020 from the most common causes of death to COVID-19. We also identified a decrease in the number of deaths and YLL from cancer and other causes among both men and women in all age groups. This may be due to several underlying processes, potentially a downward trend in overall mortality and cancer case fatality rates especially at younger ages.

As most prior studies have examined excess mortality attributable to the COVID-19 pandemic in Sweden exclusively at the beginning of the pandemic,^10^^,^^19^^,^^28^ this study provides a more comprehensive analysis of excess mortality and YLL at the individual level during the first 17 months of the COVID-19 pandemic. Similar to many Western European countries, Sweden experienced a peak (39%) in excess mortality in April 2020, followed by another peak from November 2020 to January 2021—a finding consistent with those of previous studies.^8^^,^^29^^,^^30^ Islam *et al*.^30^ estimated that 9300 (95% confidence interval: 8700–9800) excess deaths occurred in Sweden during 2020. This estimate is slightly inflated compared with our findings from individual national-level data that showed a total of 7648 confirmed crude excess deaths during 2020 compared with the crude number of deaths during 2017–19. Our results are in line with another study, which estimated that between 7040 and 7505 excess deaths occurred in Sweden in 2020, where the estimate was based on a linear trend from a longer baseline period (2010–19).^31^ The age and sex differences in excess mortality observed in this study are consistent with the findings of other studies.^30^^,^^32–34^ During the first months of the pandemic, Modig *et al*.^19^ observed excess mortality among men and women above 60 years in Stockholm County, Sweden. Hence, our study, with its extended study period of 17 months during the pandemic, confirms these authors’ findings and further identifies age and sex differences, i.e. women were mainly affected at older ages.

Based on our study findings, male deaths from COVID-19 were responsible for excess YLL during 2020, whereas YLL from CVD, cancer and other causes were lower among both men and women, compared with 2017–19. This was due to fewer deaths overall which generated less YLL. In addition, the number of excess YLL from COVID-19 was highest among men aged 65–84 years and women aged 75–110 years, while overall YLL was lower among younger men (aged 0–44 years) and women aged 0–74 years compared with the average baseline. This is consistent with findings from previous studies,^25^^,^^35^ which showed that YLL from COVID-19 was greatest in the older population (60+), the most vulnerable group. In contrast to our findings, Ebeling *et al*.^26^ concluded that COVID-19 did not replace other causes of death during 2020 because YLL from other causes of death were similar during 2019 and 2020 while Sweden experienced excess deaths. The year 2019 was an exceptional year in Sweden with unexpectedly low death rates in total^36^ (around 92 000 in both 2017 and 2018 compared with 88 000 in 2019) and in particular CVD deaths. Therefore, our studies differ in two aspects. First, the model used in Ebeling *et al*.^26^ estimated even lower mortality during 2020 compared with 2019, possibly resulting in an overestimation of the number of excess deaths during 2020. Secondly, Ebeling *et al*.^26^ compared cause-specific mortality with 2019, where there was no difference compared with 2020. However, compared with 2017 and 2018, Sweden saw considerably fewer CVD deaths in 2020 (Supplementary table S2).

COVID-19 infection drove excess mortality in Sweden during 2020, as confirmed by previous studies.^19^^,^^20^^,^^30^ Our study confirmed that there was a change in deaths from the most common causes of death during the first year of the pandemic. Both men and women experienced a considerable reduction in the number of CVD deaths, as shown by a previous study,^37^ which further contributed to the highest decrease in the number of YLL. Almost 50% of all individuals who died from COVID-19 also had CVD or cancer as a contributory cause of death. When adding these COVID-19 deaths, with CVD or cancer as contributing causes of deaths, to the deaths among CVD and cancer, the number of deaths and YLL from these causes during 2020 were more similar to that of the average baseline. In some frail and vulnerable individuals, death from COVID-19 infection—instead of from more long-term conditions such as heart failure or cancer—may simply have been the terminal event. Other factors, such as an overall ongoing decline in CVD mortality during the last decades, could play a part in this observed decline in CVD death. The identified decrease in deaths from other causes could indicate that deaths from some diseases, such as influenza^38^ or from injuries such as those from traffic accidents may have decreased during the pandemic in Sweden.^39^ However, the decrease may also reflect a normal variation or ongoing decline in overall deaths in the population.

Furthermore, overburden on the healthcare system and changes in the priority of healthcare visits in favour of caring for COVID-19 cases may have caused deficiencies in standard healthcare pathways. The fear of contracting a disease or overloading the system, which may have altered health-oriented behaviours, may have led to an increase in the number of deaths from non-COVID-19 causes compared with previous years. In particular, the number of reported heart attacks and strokes or urgent referrals for suspected cancer declined during 2020 compared with previous years,^40^ resulting in less early identification of patients with warning symptoms; this may have increased the number of avoidable deaths from heart attacks, stroke and cancer. However, this study indicates that CVD and cancer mortality decreased during 2020 compared with the average baseline. This notwithstanding, changes in healthcare-seeking behaviour may have led to long-term effects on mortality.

### Strengths and limitations

This study provides a more complete picture of the COVID-19 mortality burden in Sweden compared with previous studies by simultaneously describing excess mortality and YLL during the first 17 months of the pandemic. Study strengths include the use of both complete data from nationwide administrative registries with the linking of data for deaths, comorbidities and need of care across registries and data on cause-specific deaths and detailed life expectancy tables obtained from Statistics Sweden. However, the study has some limitations.

A limitation when estimating YLL is the use of national life tables that do not take frailty and comorbidities into account in the calculations of remaining life expectancy which causes an overestimation of YLL. This study, along with many previous studies, confirms that those who died from COVID-19 were more vulnerable and fragile than the general population, as are most who die from CVD and cancer. Not considering the differences in remaining life expectancy among those aged 70 years and older depending on the level of geriatric care has been shown to overestimate YLL attributable to COVID-19 by approximately 2 years per death.^26^ We may therefore have overestimated YLL from COVID-19, given most men and women who died from COVID-19 were older and were more burdened by comorbidities—particularly CVD and cancer—and therefore had lower life expectancy compared with the general population for which life expectancy tables are calculated.

Another limitation is that deaths from COVID-19 as reported by Statistics Sweden may be underestimated because of the misclassification of the cause of death during the early pandemic phase. Additionally, COVID-19 may have trumped most other causes of data; therefore, death from other terminally severe diseases would have been classified as COVID-19 deaths if patients had contracted the infection even if the infection had not directly caused death. Some deaths may be misclassified or indirectly related to the COVID-19 pandemic, e.g. deaths from other causes occurring in the context of an overburdened healthcare system. To offer comparability with other studies in excess mortality, we chose to include data for the full year of 2020 in our analyses, including January and February. The first case of COVID-19 in Sweden tested positive on 31 January.

Finally, all calculations of excess deaths require an estimate of expected deaths during the period of study; however, the method of choice to calculate expected deaths may greatly affect the estimate.^36^ Sweden has a downward trend in mortality rates; however, Sweden saw low mortality rates in 2019 (Supplementary table S6). Thus, the extrapolation of expected mortality rates in 2020 becomes very sensitive to the method of extrapolation. We chose a conservative estimate, using the average number of deaths in 2017–19 adjusted to the population size during 2020 as a reference, possibly underestimating excess deaths as well as YLL.

## Conclusions

We observed that Sweden experienced excess mortality and YLL from all causes of death during 2020, followed by a decrease in mortality during the first 5 months of 2021 compared with 2017–19. In 2020, men had higher excess mortality and a higher number of YLL from all-cause deaths at younger ages compared with women. Specifically, men aged 45+ years and women aged 75+ years were most affected, indicating age- and sex differences in mortality. These excess deaths and YLL were predominately from COVID-19 infections, mostly among younger men (65+). We also observed a considerable decrease in the number of deaths and YLL from CVD in both men and women in all age groups, compared with 2017–19. This may indicate a mortality displacement from the most common causes of death to deaths due to COVID-19 infection.

## Supplementary Material

ckad086_Supplementary_Data

## Data Availability

Data are available from the sources stated in the paper by request to the data providers, fulfilling legal and regulatory requirements and with permission from the Swedish Ethical Review Authority.
