# Supplementary material for: Age and sex differences in cause-specific excess mortality and years of life lost associated with COVID-19 infection in the Swedish population
Source: Eur J Public Health. 2023 Jun 1;33(5):916–22. doi: 10.1093/eurpub/ckad086 (PMC10567253; doi:10.1093/eurpub/ckad086)
Supplement: ckad086_Supplementary_Data [file ckad086_supplementary_data.docx]

**Supplementary material**

Age and sex differences in cause-specific excess mortality and years of life lost associated with COVID-19 infection in the Swedish population

**S1 Table. ICD-10 codes for cause specific mortality**

| **Comorbidity** | **ICD-10 code** |
| --- | --- |
| Cardiovascular disease | I00–I09, I11, I13, I20–I51, I63, I64 |
| Cancer | C00–C97 |

**S2 Table. Adjusted number of deaths by year, and relative difference comparing 2017, 2018 and 2019 with 2020.**

|  | **Deaths** | | | | **Difference 2020 compared with previous years** | | | |
| --- | --- | --- | --- | --- | --- | --- | --- | --- |
|  | **2017^a^**  **(column %)** | **2018 ^a^**  **(column %)** | **2019 ^a^**  **(column %)** | **2020**  **(column %)** | **2020 vs. 2017, n (%)** | **2020 vs. 2018, n (%)** | **2020 vs. 2019, n (%)** | **2020 vs. average baseline, n (%)** |
| **ALL** | | | | | | | | |
| **All deaths** | **96,851 (100)** | **95,695 (100)** | **90,537 (100)** | **98,441 (100)** | **1,590 (1.6)** | **2,746 (2.9)** | **7,904 (8.7)** | **4,080 (8.4)** |
| CVD | 26,612 (27.5) | 25,476 (26.6) | 23,187 (25.6) | 22,366 (22.7) | -4,246 (-16.0) | -3,110 (-12.2) | -821 (-3.5) | -2,725 (-10.9) |
| Cancer | 24,279 (25.1) | 23,434 (24.5) | 22,874 (25.3) | 22,523 (22.9) | -1,756 (-7.2) | -911 (-3.9) | -351 (-1.5) | -1,006 (-4.3) |
| Other | 45,961 (47.5) | 46,786 (48.9) | 44,476 (49.1) | 43,807 (44.5) | -2,154 (-4.7) | -2,979 (-6.4) | -669 (-1.5) | -1933 (-4.2) |
| COVID-19 | NA | NA | NA | 9,745 (9.9) | NA | NA | NA | 9,745 (NA) |
| *Deaths excluding COVID-19* | 96,851 (100) | 95,695 (100) | 90,537 (100) | 88,696 (90.1) | -8,155 (-8.4) | -6,999 (-7.3) | -1,841 (-2.0) | -5,665 (-6.0) |
| **MEN** | | | | | | | | |
| **All deaths** | **48,834 (100)** | **48,035 (100)** | **45,438 (100)** | **49,597 (100)** | **10 (0.0)** | **809 (1.7)** | **3,406 (7.5)** | **2,672** (5.7) |
| CVD | 13,174 (27.0) | 12,662 (26.4) | 11,395 (25.1) | 11,452 (23.1) | -2,260 (-17.2) | -1,748 (-13.8) | -481 (-4.2) | -1,229 (-9.7) |
| Cancer | 11,514 (23.6) | 10,984 (22.9) | 10,911 (24.0) | 11,872 (23.9) | -863 (-7.5) | -333 (-3.0) | -260 (-2.4) | -521 (-4.2) |
| Other | 24,146 (49.4) | 24,390 (50.8) | 23,132 (50.9) | 20,999 (42.3) | -1,338 (-5.5) | -1,582 (-6.5) | -324 (-1.4) | -852 (-3.9) |
| COVID-19 | NA | NA | NA | 5,274 (10.6) | NA | NA | NA | 5,274 (NA) |
| *Deaths excluding COVID-19* | 48,834 (100) | 48,035 (100) | 45,438 (100) | 44,323 (89.4) | -4,461 (-9.1) | -3,662 (-7.6) | -1,065 (-2.3) | -2602 (-5.5) |
| **WOMEN** | | | | | | | | |
| **All deaths** | **48,018 (100)** | **47,660 (100)** | **45,099 (100)** | **48,844 (100)** | **1,579 (3.3)** | **1,937 (4.1)** | **4,498 (10.0)** | **1,408 (3.0)** |
| CVD | 13,438 (28.0) | 12,813 (26.9) | 11,792 (26.1) | 10,914 (22.3) | -1,986 (-14.8) | -1,361 (-10.6) | -340 (-2.9) | -1,496 (-12.1) |
| Cancer | 12,765 (26.6) | 12,450 (26.1) | 11,963 (26.5) | 10,651 (21.8) | -893 (-7.0) | -578 (-4.6) | -91 (0.8) | -485 (-4.4) |
| Other | 21,815 (45.4) | 22,397 (47.1) | 21,344 (47.3) | 22,808 (46.7) | -816 (-3.7) | -1,398 (-6.2) | -345 (-1.6) | -1,081 (-4.5) |
| COVID-19 | NA | NA | NA | 4,471 (9.2) | NA | NA | NA | 4,471 (NA) |
| *Deaths excluding COVID-19* | 48,018 (100) | 47,660 (100) | 45,099 (100) | 44,373 (90.8) | -3,695 (-7.7) | -3,337 (-7.0) | -776 (-1.7) | -3,063 (-6.5) |

^a^ Adjusted to the population size of 2020

**S3 Table. Adjusted^a^ years of life lost by year, and relative difference comparing 2017, 2018 and 2019 with 2020.**

|  | **Years of life lost** | | | | **Difference 2020 compared with previous years** | | | |
| --- | --- | --- | --- | --- | --- | --- | --- | --- |
|  | **2017^a^**  **(column %)** | **2018^a^**  **(column %)** | **2019^a^**  **(column %)** | **2020**  **(column %)** | **2020 vs. 2017, n (%)** | **2020 vs. 2018, n (%)** | **2020 vs. 2019, n (%)** | **2020 vs. average, n (%)** |
| **ALL** | | | | | | | | |
| **All deaths** | **945,481 (100)** | **946,333 (100)** | **920,891 (100)** | **960,305 (100)** | **14,823 (1.5)** | **13,972 (1.5)** | **39,413 (4.1)** | **22 737 (2.4)** |
| CVD | 195,215 (20.6) | 191,932 (20.3) | 176,287 (19.1) | 168,000 (17.5) | -27,215 (-16.2) | -23,932 (-14.2) | -8,287 (-4.9) | -19,810 (-10.5) |
| Cancer | 299,629 (31.7) | 289,735 (31.7) | 288,904 (31.4) | 278,224 (29.0) | -21,405 (-7.7) | -11,511 (-4.1) | -10,681 (-3.8) | -14,533 (-5.0) |
| Other | 450,637 (47.7) | 464,666 (47.7) | 455,700 (49.5) | 438,930 (45.7) | -11,708 (-2.7) | -25,736 (-5.9) | -16,770 (-3.8) | -18,071 (-4.0) |
| COVID-19 | NA | NA | NA | 75,151 (7.8) | NA | NA | NA | 75,152 (NA) |
| *Deaths excluding COVID-19* | 945,481 (100) | 946,333 (100) | 920,891 (100) | 885,153 (92.2) | -60,328 (-6.8) | -61,180 (-6.9) | -35,738 (-4.0) | -52,415 (-5.6) |
| **MEN** | | | | | | | | |
| **All deaths** | **503,615 (100)** | **505,943 (100)** | **489,129 (100)** | **518,703 (100)** | **15,088 (2.9)** | **12,760 (2.5)** | **29,573 (5.7)** | **19,141 (3.8)** |
| CVD | 112,835 (22.4) | 110,268 (22.4) | 101,530 (20.8) | 98,280 (18.9) | -14,555 (-14.8) | -11,989 (-12.2) | -3,250 (-3.3) | -9,931 (-9.2) |
| Cancer | 145,247 (28.8) | 141,064 (28.8) | 138,276 (28.3) | 135,557 (26.1) | -9,690 (-7.1) | -5,507 (4.1) | -2,720 (-2.0) | -5,972 (-4.2) |
| Other | 245,533 (48.8) | 254,610 (48.8) | 249,323 (51.0) | 241,483 (46.6) | -4,051 (-1.7) | -13,128 (-5.4) | -7,841 (-3.2) | -8,339 (-3.3) |
| COVID-19 | NA | NA | NA | 43,384 (8.4) | NA | NA | NA | 43,383 (NA) |
| *Deaths excluding COVID-19* | 503,615 (100) | 505,943 (100) | 489,129 (100) | 475,319 (91.6) | -28,295 (-6.0) | -30,624 (-6.4) | -13,810 (-2.9) | -24,242 (-4.9) |
| **WOMEN** | | | | | | | | |
| **All deaths** | **441,867 (100)** | **440,390 (100)** | **431,762 (100)** | **441,602 (100)** | **-265 (-0.1)** | **1,212 (0.3)** | **9,840 (2.2)** | **3,596 (0.8)** |
| CVD | 82,380 (18.6) | 81,664 (18.6) | 74,757 (17.3) | 69,720 (15.8) | -12,660 (-18.2) | -11,944 (-17.1) | -5,037 (-7.2) | -9,880 (-12.4) |
| Cancer | 154,382 (34.9) | 148,670 (34.9) | 150,628 (34.9) | 142,667 (32.3) | -11,715 (-8.2) | -6,004 (-4.2) | -7,961 (-5.6) | -8,560 (-5.7) |
| Other | 205,104 (46.4) | 210,056 (46.4) | 206,377 (47.8) | 197,447 (44.7) | -7,657 (-3.9) | -12,609 (-6.4) | -8,930 (-5.6) | -9,732 (-4.7) |
| COVID-19 | NA | NA | NA | 31,768 (7.2) | NA | NA | NA | 31,768 (NA) |
| *Deaths excluding COVID-19* | 441,867 (100) | 440,390 (100) | 431,762 (100) | 409,834 (92.8) | -32,033 (-7.8) | -30,556 (-7.5) | -21,928 (-5.4) | -28,172 (-6.4) |

^a^ Adjusted to the population size of 2020

The mean life expectancy during the 2017–2019 period was used to calculate YLL for 2020.

**S4 Table. Crude monthly excess mortality from baseline using historical average for all deaths during the first five months of 2021 in total, men and women**

| **Year 2021** | **Total** | | | **Men** | | | **Women** | | |
| --- | --- | --- | --- | --- | --- | --- | --- | --- | --- |
|  | **Average**  **2017–2019** | **Observed mortality 2021** | **Excess deaths from baseline using historical average (n, %)** | **Average**  **2017–2019** | **Observed mortality 2021** | **Excess deaths from baseline using historical average (n, %)** | **Average**  **2017–2019** | **Observed mortality 2021** | **Excess deaths from baseline using historical average (n, %)** |
| Jan | 8,806 | 10,336 | 1,530 (17.4) | 4,295 | 5,249 | 954 (22.2) | 4,511 | 5,087 | 576 (12.8) |
| Feb | 7,936 | 7,600 | -336 (-4.2) | 3,825 | 3,883 | 58 (1.5) | 4,111 | 3,717 | -394 (-9.6) |
| Mar | 8,476 | 7,853 | -623 (-7.3) | 4,108 | 4,005 | -103 (-2.5) | 4,368 | 3,848 | -520 (-11.9) |
| Apr | 7,617 | 7,355 | -262 (-3.4) | 3,724 | 3,766 | 42 (1.1) | 3,892 | 3,589 | -303 (-7.8) |
| May | 7,178 | 7,246 | 68 (0.9) | 3,561 | 3,752 | 191 (5.4) | 3,617 | 3,494 | -123 (-3.4) |
| **Total** | **40,013** | **40,390** | **377 (0.9)** | **19,513** | **20,655** | **1,142 (5.9)** | **20,499** | **19,735** | **-764 (-3.7)** |

**S5 Table. Characteristics of individuals who died with COVID-19 and non-COVID-19 death during 2020.**

| **Characteristics** | **COVID-19 death** | | | **Non-COVID-19 death** | | |
| --- | --- | --- | --- | --- | --- | --- |
|  | **All** | **Men** | **Women** | **All** | **Men** | **Women** |
| **n** | **9,745** | **5,274** | **4,471** | **88,696** | **44,323** | **44,373** |
| Age, mean (SD) | 83.9 (10.4) | 82.0 (10.6) | 86.1 (9.6) | 80.9 (13.61) | 78.5 (14.0) | 83.3 (12.8) |
| **Age group, n (%)** | | | | | | |
| 0 to 44 | 52 (0.5) | 31 (0.6) | 21 (0.5) | 2,068 (2.3) | 1,369 (3.1) | 699 (1.6) |
| 45 to 64 | 447 (4.6) | 330 (6.3) | 117 (2.6) | 7,287 (8.2) | 4,474 (10.1) | 2,813 (6.3) |
| 65 to 74 | 1,067 (10.9) | 743 (14.1) | 324 (7.2) | 13,662 (5.4) | 8,048 (18.2) | 5,614 (12.7) |
| 75 to 84 | 3,082 (31.6) | 1,853 (35.1) | 1,229 (27.5) | 26,084 (29.4) | 14,409 (32.5) | 11,675 (26.3) |
| 85 to 110 | 5,097 (52.3) | 2,317 (43.9) | 2,780 (62.2) | 39,595 (44.6) | 16,023 (36.2) | 23,572 (53.1) |
| **Contributory cause of death** | | | | | | |
| CVD or Cancer | 4,469 (45.9) | 2,578 (48.9) | 1,891 (42.3) | 28,824 (32.5) | 15,289 (34.5) | 13,535 (30.5) |

**S6 Table. Crude monthly excess mortality in 2020 compared to the baseline 2017 and 2019 using historical average in all ages for all deaths and specific cause of deaths (CVD, cancer, other causes and all deaths excluding COVID-19 deaths) in men and women, presented as n (%)**

| **Month** | **All deaths** | | | **CVD deaths** | | | **Cancer deaths** | | | **Other deaths** | | | **Deaths excluding**  **COVID-19** | | |
| --- | --- | --- | --- | --- | --- | --- | --- | --- | --- | --- | --- | --- | --- | --- | --- |
|  | **Total** | **Men** | **Women** | **Total** | **Men** | **Women** | **Total** | **Men** | **Women** | **Total** | **Men** | **Women** | **Total** | **Men** | **Women** |
| Jan | -483 (-5.5) | -204 (-4.8) | -279 (-6.2) | -306 (-13.0) | -130 (-11.1) | -176 (-14.8) | 17 (0.9) | -32 (-3.0) | 49 (5.3) | -194 (-4.4) | -42 (-2.0) | -152 (-6.4) | -482 (-5.5) | -204 (-4.8) | -279 (-6.2) |
| Feb | -411 (-5.2) | -84 (-2.2) | -327 (-7.9) | -238 (-11.1) | -81 (-7.6) | -157 (-14.6) | 63 (3.6) | 55 (6.2) | 8 (0.9) | -236 (-5.8) | -59 (-3.2) | -176 (-8.1) | -411 (-5.2) | -84 (-2.2) | -327 (-7.9) |
| Mar | 69 (0.8) | 163 (4.0) | -94 (-2.1) | -281 (-12.4) | -131 (-11.7) | -150 (-13.1) | 40 (2.0) | 18 (1.7) | 22 (2.4) | -24 (-0.6) | 79 (4.1) | -104 (-4.5) | -266 (-3.1) | -34 (-0.8) | -232 (-5.3) |
| Apr | 2,968 (39) | 1,678 (45.0) | 1,291 (33.2) | -76 (-3.7) | -1 (-0.1) | -76 (-7.3) | -44 (-2.4) | -31 (-3.3) | -13 (-1.4) | 284 (7.6) | 156 (8.8) | 129 (6.6) | 164 (2.2) | 124 (3.3) | 41 (1.0) |
| May | 1,781 (24.8) | 957 (26.9) | 824 (22.8) | -81 (-4.1) | -30 (-3.0) | -51 (-5.3) | 30 (1.7) | 20 (2.1) | 10 (1.2) | 69 (2.0) | 50 (3.1) | 18 (1.0) | 18 (0.3) | 40 (1.1) | -22 (-0.6) |
| June | 785 (11.3) | 530 (15.3) | 255 (7.4) | -59 (-3.2) | 10 (1.1) | -69 (-7.6) | -69 (-3.8) | -10 (-1.1) | -59 (-6.7) | 49 (1.5) | 51 (3.2) | -2 (-0.1) | -79 (-1.1) | 51 (1.5) | -130 (-3.8) |
| July | -5 (-0.1) | 57 (1.6) | -62 (-1.7) | -173 (-9.4) | -86 (-9.4) | -88 (-9.5) | -24 (-1.3) | 22 (2.2) | -46 (-5.1) | -73 (-2.2) | -26 (-1.6) | -47 (-2.6) | -270 (-3.8) | -90 (-2.6) | -180 (-5.0) |
| Aug | -5 (-0.1) | 23 (0.7) | -28 (-0.8) | -118 (-6.5) | -48 (-5.4) | -70 (-7.5) | 35 (1.9) | 23 (2.3) | 12 (1.4) | -4 (-0.1) | 1 (0.1) | -5 (-0.3) | -87 (-1.2) | -24 (-0.7) | -63 (-1.8) |
| Sep | -108 (-1.6) | -102 (-3.0) | -6 (-0.2) | -77 (-4.3) | -65 (-7.0) | -12 (-1.4) | -81 (-4.4) | -34 (-3.5) | -47 (-5.4) | -2 (-0.1) | -35 (-2.3) | 33 (1.9) | -160 (-2.3) | -134 (-3.9) | -26 (-0.8) |
| Oct | -219 (-2.9) | -11 (-0.3) | -208 (-5.5) | -126 (-6.4) | -8 (-0.8) | -118 (-12.5) | -3 (-0.2) | 34 (3.3) | -38 (-4.0) | -229 (-6.4) | -120 (-7.0) | -110 (-5.9) | -359 (-4.8) | -93 (-2.5) | -266 (-7.1) |
| Nov | 964 (13.2) | 586 (16.2) | 378 (10.2) | -88 (-4.5) | 2 (0.2) | -89 (-9.1) | -55 (-2.9) | -17 (-1.7) | -38 (-4.2) | -17 (-0.5) | 19 (1.1) | -36 (-2.0) | -160 (-2.2) | 3 (0.1) | -163 (-4.4) |
| Dec | 2,312 (28.7) | 1,326 (33.5) | 985 (24.0) | -123 (-5.6) | -22 (-2.1) | -100 (-9.1) | -3 (-0.1) | 20 (2.0) | -22 (-2.4) | 120 (3.1) | 93 (4.9) | 28 (1.3) | -5 (-0.1) | 90 (2.3) | -95 (-2.3) |
| **Total** | **7,648 (8.4)** | **4,919 (11.0)** | **2,729 (5.9)** | **1,746 (-7.2)** | **-590 (-4.9)** | **-1,156 (-9.6)** | **-94 (-0.4)** | **68 (0.6)** | **-162 (-1.5)** | **-257 (-0.6)** | **167 (0.8)** | **-424 (-1.8)** | **-2,097 (-2.3)** | **-355 (-0.8)** | **-1,742 (-3.8)** |

**S7 Table. Sensitivity analysis of adjusted^a^ excess deaths and years of life lost for CVD, cancer with COVID-19 cases calculated with and without CVD/ cancer as contributing cause of death, by men and women.**

|  | **Average baseline 2017–2019** | | | **2020** | | | **Difference compared with average baseline** | |
| --- | --- | --- | --- | --- | --- | --- | --- | --- |
|  | **Deaths^a^**  **(column %)** | **YLL^a^**  **(column %)** | **YLL/death** | **Deaths**  **(column %)** | **YLL**  **(column %)** | **YLL/death** | **Deaths, n (%)** | **YLL, n (%)** |
| **MEN** | | | | | | | | |
| **All deaths** | **46,925 (100)** | **499,562 (100)** | **11.2** | **49,597 (100)** | **518,703 (100)** | **10.5** | **2,672** (5.7) | **19,141 (3.8)** |
| CVD | 12,681 (27.0) | 108,211 (21.7) | 8.5 | 11,452 (23.1) | 98,280 (18.9) | 8.6 | -1,229 (-9.7) | -9,931 (-9.2) |
| CVD and Covid-19^b^ | 12,681 (27.0) | 108,211 (21.7) | 8.5 | 13,664 (27.6) | 113,453 (21.9) | 8.3 | 983 (7.8%) | 5,242 (4.8) |
| Cancer | 12,393 (26.4) | 141,529 (28.3) | 11.4 | 11,872 (23.9) | 135,557 (26.1) | 11.4 | -521 (-4.2) | -5,972 (-4.2) |
| Cancer and Coivd-19^c^ | 12,393 (26.4) | 141,529 (28.3) | 11.4 | 12,494 (25.2) | 140,374 (27.1) | 11.2 | 101 (0.8) | -1,155,5 (-0.8) |
| COVID-19, all | NA | NA | NA | 5,274 (10.6) | 43,384 (8.4) | 8.2 | 5,274 (NA) | 43,383 (NA) |
| COVID-19 **without** CVD or cancer  as contributing cause of death |  |  |  | 2,440 (4.9) | 23,393 (4.5) | 9,6 |  |  |
| COVID-19 **with** CVD or cancer  as contributing cause of death |  |  |  | 2,834 (5.7) | 19,990 (3.9) | 7,1 |  |  |
| **Women** | | | | | | | | |
| **All deaths** | **47,436 (100)** | **438,006 (100)** | **9.5** | **48,844 (100)** | **441,602 (100)** | **9.0** | **1,408 (3.0)** | **3,596 (0.8)** |
| CVD, all | 12,410 (26.2) | 79,600 (18.2) | 6.4 | 10,914 (22.3) | 69,720 (15.8) | 6.4 | -1,496 (-12.1) | -9,880 (-12.4) |
| CVD and Covid-19^b^ | 12,410 (26.2) | 79,600 (18.2) | 6.4 | 12,614 (25.8) | 79,974 (18.1) | 6.3 | 204 (1.6) | 374 (0.5) |
| Cancer, all | 11,136 (23.4) | 151,227 (34.5) | 13.6 | 10,651 (21.8) | 142,667 (32.3) | 13.4 | -485 (-4.4) | -8,560 (-5.7) |
| Cancer and Coivd-19^c^ | 11,136 (23.4) | 151,227 (34.5) | 13.6 | 10,949 (22.4) | 145,436 (32.9) | 13.3 | -187 (-1.7) | -5,791 (-3.8) |
| COVID-19, all | NA | NA | NA | 4,471 (9.2) | 31,768 (7.2) | 7.1 | 4,471 (NA) | 31,768 (NA) |
| COVID-19 **without** CVD or cancer  as contributing cause of death |  |  |  | 2,473 (5.6) | 18,745 (4.2) | 7.6 |  |  |
| COVID-19 **with** CVD or cancer  as contributing cause of death |  |  |  | 1,998 (4.1) | 13,023 (2.9) | 6.5 |  |  |

^a^ Adjusted to the population size of 2020

^b^ COVID-19 cases with CVD as contributing cause of death, any position

^c^ COVID-19 cases with cancer as contributing causes of death, any position

**Figure S1. Crude monthly deaths from all-causes and deaths excluding COVID-19 during 2020 and all-cause deaths during the first five months of 2021, compared with average monthly deaths from all-causes during 2017–2019, in the Swedish population.**

**Figure S2. Adjusted^a^ number of deaths and years of life lost for all-causes and for selected causes of deaths during 2020 for men, by one-year age groups**

Abbreviations: CVD: Cardiovascular disease

^a^ Adjusted to the population size of 2020

**Figure S3. Adjusted number of deaths and years of life lost for all-causes and for selected causes of deaths during 2020 for women, by one-year age groups.**

Abbreviations: CVD: Cardiovascular disease

^a^ Adjusted to the population size of 2020
